# Employing PCBTDPP as an Efficient Donor Polymer for High Performance Ternary Polymer Solar Cells

**DOI:** 10.3390/polym11091423

**Published:** 2019-08-29

**Authors:** Binrui Xu, Gopalan Saianand, V. A. L. Roy, Qiquan Qiao, Khan Mamun Reza, Shin-Won Kang

**Affiliations:** 1 School of Electronics Engineering, College of IT Engineering, Kyungpook National University, Daegu 41566, Korea; 2 Global Innovative Center for Advanced Nanomaterials, School of Engineering, Faculty of Engineering and Built Environment, The University of Newcastle, Callaghan, NSW 2308, Australia; 3 Department of Materials Science and Engineering, City University of Hong Kong, Tat Chee Avenue, Kowloon 999077, Hong Kong, China; 4 Department of Electrical Engineering and Computer Science, South Dakota State University, Brookings, SD 570007, USA

**Keywords:** polymer solar cells, low-bandgap, ternary, PCBTDPP

## Abstract

A compatible low-bandgap donor polymer (poly[*N*-90-heptadecanyl-2,7carbazole-alt-3,6-bis(thiophen-5-yl)-2,5-dioctyl-2,5-dihydropyrrolo[3,4]pyrrole-1,4-dione], PCBTDPP) was judicially introduced into the archetypal poly(3-hexylthiophene):[6,6]-phenyl-C_61_-butyric acid methyl ester (P3HT:PC_61_BM) photoactive system to fabricate highly efficient ternary based bulk heterojunction polymer solar cells (PSCs). The PCBTDPP ternary-based PSC with optimal loading (0.2 wt.%) displayed outstanding performance with a champion power conversion efficiency (PCE) of 5.28% as compared to the PCE (4.67%) for P3HT:PC_61_BM-based PSC (reference). The improved PCE for PCBTDPP ternary-based PSC can be mainly attributed to the incorporation of PCBTDPP into P3HT:PC_61_BM that beneficially improved the optical, morphological, electronic, and photovoltaic (PV) performance. This work instills a rational strategy for identifying components (donor/acceptor (D/A) molecules) with complementary beneficial properties toward fabricating efficient ternary PSCs.

## 1. Introduction

**Figure 1:**
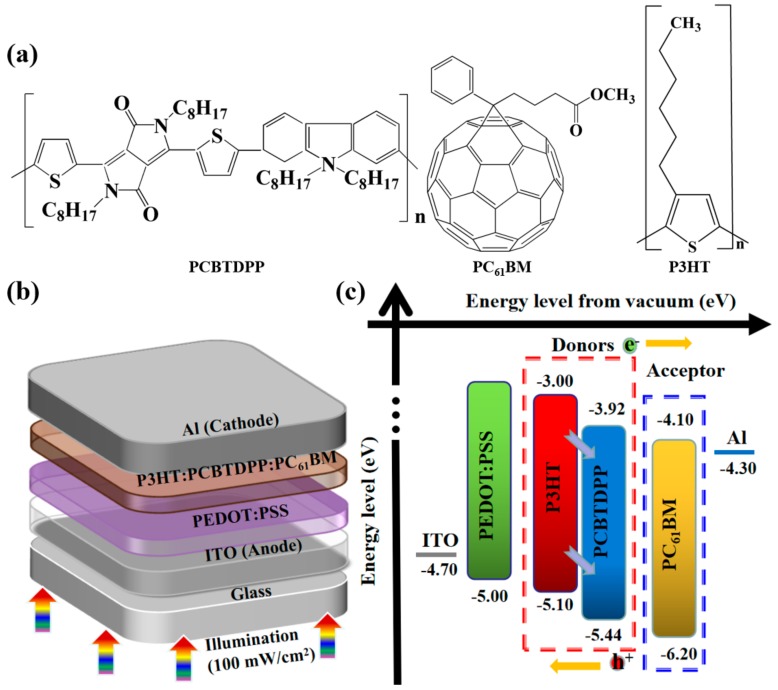
(**a**) The chemical structures of poly[*N*-90-heptadecanyl-2,7carbazole-alt-3,6-bis(thiophen-5-yl)-2,5-dioctyl-2,5-dihydropyrrolo[3,4]pyrrole-1,4-dione] (PCBTDPP), poly(3-hexylthiophene):[6,6] (P3HT), and phenyl-C61-butyric acid methyl ester (PC61BM), (**b**) device geometry, and (**c**) energy band levels of the studied components. ITO = indium tin oxide; poly (3,4-ethylene dioxythiophene): poly (styrene sulfonate) = PEDOT:PSS.

Recently, polymer solar cells (PSCs) have triggered stupendous attention owing to their cost-competitiveness, mechanical flexibility, lightweight nature, and compatibility for large area deposition [1,2,3,4,5,6,7]. One of the most widely used donor/acceptor (D/A) photoactive blend systems is poly(3-hexylthiophene):[6,6]-phenyl-C_61_-butyric acid methyl ester (P3HT:PC_61_BM) [8,9,10,11,12]. So far, the power conversion efficiency (PCE) of PSCs with the conventional P3HT:PC_61_BM system have achieved 3–5% [13,14,15,16]. However, further development on P3HT:PC_61_BM-based PSCs is highly warranted to overcome the low short-circuit current density (*J_sc_*), which can be improved by tailoring the relatively narrow absorption range due to the wide-bandgap nature of the P3HT. To widen the light absorption capability of the P3HT:PC_61_BM and to further improve photovoltaic (PV) performance, numerous strategies have been widely demonstrated, such as synthesizing new low-bandgap materials, employing tandem PSCs, doping suitable D/A molecules, etc. [17,18,19,20,21]. Among them, adopting ternary-based PSCs can be considered one of the most promising strategies to manipulate/fine-tune the PV performance parameters of the PSCs [22,23]. Recently, a ternary photoactive blend system (D/D/A, D/A/A) featuring a low-bandgap material with broad absorption spectra has been realized as a potential strategy to improve optical absorption, optimize the exciton separation, and charge carrier extraction in active layer with an aim of enhancing the *J_sc_* and other performance parameters of PSCs [24,25,26,27]. Herein, we explored a new kind of unexplored low-bandgap polymer, poly[*N*-9-hepta-decanyl-2,7-carbazole-alt-3,6-bis-(thiophen-5-yl)-2,5-dioctyl-2,5-dihydropyrrolo[3,4]pyrrole-1,4-dione] (PCBTDPP, Figure 1a), as an efficient donor into P3HT:PC_61_BM active system to construct high-efficiency ternary PV devices with the device architecture (Figure 1b): indium tin oxide (ITO) glass/poly (3,4-ethylene dioxythiophene): poly (styrene sulfonate) (PEDOT:PSS)/binary or ternary photoactive blend system/Al. Figure 1c depicts the energy level bands of components involved, and their energy levels are derived from the relevant reports [28,29,30,31]. 

PCBTDPP was primarily chosen for two main reasons. Firstly, PCBTDPP is a low-bandgap (1.52 eV) donor with good stability, high mobility and complementary absorption spectrum (300–800 nm) to P3HT [29]. In addition, the highest occupied molecular orbital (HOMO) and the lowest unoccupied molecular orbital (LUMO) energy levels of PCBTDPP are comparable to that of P3HT and PC_61_BM. Secondly, PCBTDPP has good compatibility and solubility in the photoactive blend, P3HT:PC_61_BM (1,2-dichlorobenzene (DCB)), which is expected to influence the optoelectronic properties of the ternary-based PSC. Earlier it was reported that PCBTDPP:PC_70_BM based PSC witnessed an inferior PCE of 1.6% [32]. The beneficial optoelectronic properties of PCBTDPP in P3HT:PC_61_BM based ternary PSC has not been harnessed so far. To the best of our knowledge, the role and influence of PCBTDPP into P3HT:PC_61_BM on the optical, structural, morphology and PV performance has not been studied in detail. Hence, in this contribution, we clarify the relevant improvements on the optoelectronic properties, as well as their corresponding PV performance. Consequently, the ternary PSC-based on optimal loading of PCBTDPP (0.2 wt.%) into P3HT: PC_61_BM witnessed a high PCE of 5.28%, while the fabricated binary P3HT:PC_61_BM-based PSC only displayed a PCE of 4.67%.

## 2. Experimental

### 2.1. Materials

P3HT and PC_61_BM were sourced from Lumtec, Taipei Taiwan, China. PCBTDPP and DCB were bought from Sigma-Aldrich, Seoul, Korea. Low conductive PEDOT:PSS (AI 4083) and highly conductive PEDOT:PSS (PH 500) were obtained from Baytron, H.C. Starck, Newton, MA, USA. 

### 2.2. Photoactive Ink Formulation

P3HT, PCBTDPP and PC_61_BM were blended together in host solvent, DCB with a varied weight ratio of PCBTDPP (0 to 0.3%), and stirred at 60 °C overnight. The weight ratio of PCBTDPP was chosen based on the weight ratio of P3HT.

### 2.3. Fabrication of PSCs

The ITO glass substrates (3 cm × 3 cm) were washed by acetone, methanol, and 2-propanol and UV ozone cured for 30 min based on our previous works [33,34,35]. Then, the prepared ITO glasses were dried by nitrogen gun and heated at 150 °C for 10 min. Two variants (low and highly conductive) of PEDOT:PSS (AI 4083 and PH 500) were independently deposited on the ITO glasses at 4000 rpm for 30 s and baked at 150 °C for 10 min, respectively. The as-prepared photoactive ink solution was spun onto PEDOT:PSS layer at 1200 rpm for 60 s. To complete the device fabrication, an Al electrode was thermally evaporated in high vacuum (1.5 × 10^−5^ Torr) to form an active area of 9 mm^2^. 

### 2.4. Characterization

The photocurrent density-voltage (*J-V*) parameters were recorded using a 2400 source meter (Keithley, Seoul, Korea) under solar simulator (XES-300S1, SAN-EI Electric Corporation, Osaka, Japan) with 100 mW/cm^2^ (AM 1.5G). Ultraviolet-visible (UV-vis) absorption was collected a UV-vis spectrometer (Shimadzu Corporation, Kyoto, Japan), and photoluminescence (PL) spectra were measured by a PL spectrophotometer (Spectra pro 2150i, Acton Research Corporation, Greer, MA, USA). The morphological properties of active layers were recorded by an Agilent 5500 atomic force microscope (AFM) (Agilent Technologies, Santa Clara, CA, USA). The external quantum efficiency (EQE) data was examined by a lock-in amplifier (Newport Corporation, Irvine, CA, USA).

## 3. Results and Discussion

**Figure 2:**
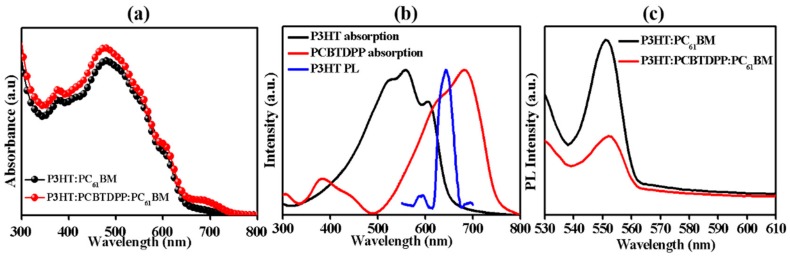
(**a**) UV-vis absorption of P3HT:PC_61_BM and P3HT:PCBTDPP(0.2 wt.%):PC_61_BM, (**b**) normalized absorption of as-casted P3HT and PCBTDPP and the PL of the P3HT, and (**c**) PL of P3HT:PC_61_BM and P3HT:PCBTDPP(0.2 wt.%):PC_61_BM thin films.

Figure 2a showed the UV-vis absorbance of as-deposited P3HT:PC_61_BM and P3HT:PCBTDPP(0.2 wt.%):PC_61_BM active layers. Figure 2b showed the UV-vis absorbance of as-casted P3HT and PCBTDPP thin films, along with their respective PL spectrum of P3HT. The P3HT:PCBTDPP(0.2 wt.%):PC_61_BM film exhibited significantly improved absorption potential in the broadband range with high intensity, which is attributed to the complementary absorption spectra capability of PCBTDPP film (Figure 2b) [36,37]. PCBTDPP displayed strong light-absorbing ability from 615 nm to 700 nm, and the main absorption peaks of PCBTDPP was centered at 681 nm. Especially, the broaden absorption range between 720 nm and 800 nm in P3HT:PCBTDPP(0.2 wt.%):PC_61_BM blend system implied that the lower energy photons could be absorbed by PCBTDPP. It is believed that good photon harvesting and efficient charge collection are the primary factors that help to enhance *J_sc_*. Results from Figure 2b indicated that the PCBTDPP absorption range covered the PL spectrum of P3HT, and could result in Forster resonance energy transfer (FRET) between P3HT and PCBTDPP [38]. The exciton generated in P3HT or PCBTDPP can be either dissociated at the D/A interfaces or directly transferred from P3HT to PCBTDPP via FRET before dissociation [39]. The HOMO and LUMO energy levels of P3HT are −5.10 eV and −3.00 eV, while PCBTDPP shows the HOMO of −5.44 eV and LUMO of −3.92 eV. The differences between LUMO levels of P3HT/PCBTDPP and LUMO of PC_61_BM are 1.10 eV and 0.18 eV, respectively, which can provide improved exciton dissociation and enhanced charge transport in P3HT:PCBTDPP(0.2 wt.%):PC_61_BM layer. Herein, the energy level of PCBTDPP was ideally placed in conjunction with P3HT and PC_61_BM for a broadened absorption potential and enhanced charge carrier transport. Additionally, the PL (Figure 2c) for two active layers was acquired under 550 nm light excitation. The peak of P3HT PL intensity at ~552 nm was considerably quenched in P3HT:PCBTDPP(0.2 wt.%):PC_61_BM as compared to P3HT:PC_61_BM, suggesting an effective exciton dissociation in the fabricated ternary blend system [40].

**Figure 3:**
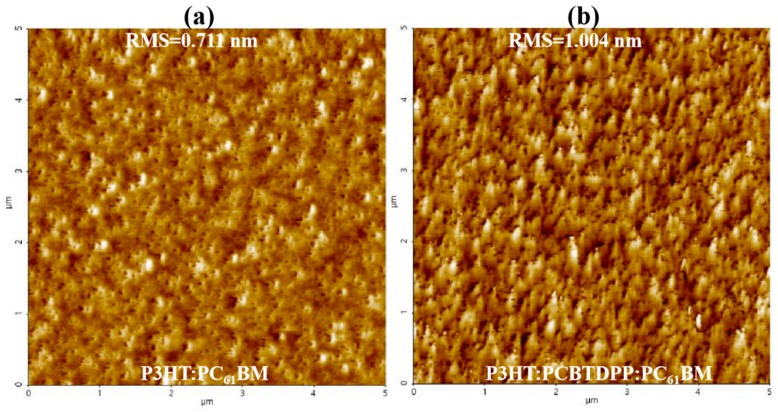
Atomic force microscope (AFM) topography images of the (**a**) binary and (**b**) ternary blend active layer coated atop ITO.

The morphological aspects of the active blend system highly influence the performance of the PV device. To probe the morphological aspects of P3HT:PC_61_BM and P3HT:PCBTDPP(0.2 wt.%):PC_61_BM, tapping mode AFM images (5 µm × 5 µm) were recorded and displayed in Figure 3a,b. Desirable nanoscale phase separation with uniform coverage and fiber-like structure (P3HT chains) with an excellent bicontinuous interpenetrating network were clearly observed for P3HT:PCBTDPP(0.2 wt.%):PC_61_BM film, resulting from effective exciton dissociation, improved charge transport and reduced charge recombination [41,42]. The root mean square (RMS) roughness of binary and ternary active layers are 0.711 nm and 1.004 nm, respectively. The rougher ternary active layer could provide a larger interface contact between the P3HT:PCBTDPP(0.2 wt.%):PC_61_BM film and Al, benefitting charge collection as well as a rapid pathway for charge transport, which leads to the quenching of PL response [43]. In addition, the rougher morphology might also induce stronger internal reflection, thus enhancing light absorbance of the active blend system. The change in RMS values indicated that the doping of PCBTDPP into P3HT:PC_61_BM allowed modification of the miscibility of P3HT and PC_61_BM [44,45].

**Figure 4:**
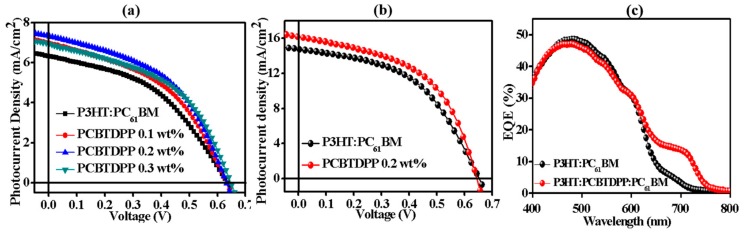
*J-V* curves of the devices based on (**a**) PEDOT:PSS (AI 4083) and (**b**) PEDOT:PSS (PH 500) with 5% DMSO and (**c**) EQE profiles of the devices based on P3HT:PC_61_BM and P3HT:PCBTDPP(0.2 wt.%):PC_61_BM.

The *J-V* characteristics and corresponding PV performance parameters of the binary (P3HT:PC_61_BM) and ternary (P3HT:PCBTDPP(0.2 wt.%):PC_61_BM) based PSCs were shown in Figure 4a,b, Table 1 and Table 2, respectively. By utilizing PEDOT:PSS (AI 4083) as a hole transport layer (HTL), the binary device showed a low PCE of 1.75%. On the contrary, the ternary P3HT:PCBTDPP(0.2 wt.%):PC_61_BM based device achieved the PCE of 2.09%. With the aim of improving the device performance, we utilized PEDOT:PSS (PH 500) with 5% DMSO as HTL, the ternary PSCs achieved champion PCE of 5.28% with a high *J_sc_* of 16.15 mA/cm^2^, while the PCE and *J_sc_* of the binary device were 4.67% and 14.75 mA/cm^2^, respectively [46]. The high *J_sc_* obtained in this work may be possibly due to doping of DMSO into PEDOT:PSS (PH 500) which would improve the conductivity of the PEDOT:PSS layer significantly. The increased PCE is also attributed to the enhanced *J_sc_*, which arises from the enhanced optical absorption, modified morphology, and improved exciton dissociation through the judicial inclusion of PCBTDPP in the ternary active system. In order to investigate the absorption efficiency and charge carriers’ dynamics, EQE spectra were recorded and shown in Figure 4c. Compared with the P3HT:PC_61_BM (binary) based device, the P3HT:PCBTDPP(0.2 wt.%):PC_61_BM (ternary) device exhibited improved EQE spectrum in a broad wavelength range (590 ~ 800 nm). In light of the optical properties, the EQE improvement mainly originates from the increased photon harvesting and the efficient exciton migration by FRET [47].

Additionally, in order to highlight the functionality of PCBTDPP in the ternary PSCs, we compared with some recent ternary PSCs based on the P3HT:PCBM:X (X: active component, D or A) (Table 3). In comparison with the previous reports, our ternary PSC-based on P3HT:PCBTDPP(0.2 wt.%):PC_61_BM achieved the best PCE of 5.28%. This comparison clearly indicates that introducing PCBTDPP into P3HT:PC_61_BM active system is one of the most efficient strategies to improve the optoelectronic and PV performance of PSCs. 

## 4. Conclusions

In conclusion, we utilized a compatible low-bandgap polymer, PCBTDPP into a standard P3HT:PC_61_BM active system to fabricate efficient ternary PSC with a high PCE of 5.28% as compared to that of the device based on binary P3HT:PC_61_BM (4.67%). The improvement in the device performance could be mainly attributed to the choice of complementary donor polymer (high mobility, extended absorption), improved exciton migration/dissociation and energy transfer through the judicial incorporation of PCBTDPP into P3HT:PC_61_BM. Our results, based on this work, clearly demonstrate the incorporation of a compatible low-bandgap donor into active layer served as an efficient and simple strategy to improve the PV performance of PSCs. Therefore, future work should focus on identifying new kinds of suitable D/A molecules with complementary material properties toward realizing highly efficient ternary-based PSCs. 

## Figures and Tables

**Table 1:** Summary of photovoltaic (PV) performances of the devices based on low conductive PEDOT:PSS (AI 4083).

PEDOT:PSS (AI 4083)	*V_oc_* (V)	*J_sc_* (mA/cm^2^)	FF	PCE (%)
P3HT:PC_61_BM	0.63	6.32	0.43	1.75
PCBTDPP 0.1 wt.%	0.63	6.71	0.44	1.86
PCBTDPP 0.2 wt.%	0.63	6.94	0.46	2.09
PCBTDPP 0.3 wt.%	0.63	6.81	0.45	1.98

**Table 2:** Summary of PV performances of the devices based on highly conductive PEDOT:PSS (PH 500) with 5% DMSO.

PEDOT:PSS (PH 500)	*V_oc_* (V)	*J_sc_* (mA/cm^2^)	FF	PCE (%)
P3HT:PC_61_BM	0.65	14.75	0.48	4.67
PCBTDPP 0.2 wt.%	0.64	16.15	0.50	5.28

**Table 3:** Summary of the PV performances of recent ternary polymer solar cells (PSCs).

Photoactive Layer	*V_oc_* (V)	*J_sc_* (mA/cm^2^)	FF	PCE (%)	Ref
P3HT:PC_61_BM:CdSe	0.60	8.15	0.62	3.05	[48]
P3HT:PC_61_BM:PCPDTBT	0.62	8.02	0.55	2.8	[49]
P3HT:PC_61_BM:Si-PCPDTBT	0.59	11	0.62	4.0	[50]
P3HT:PC_61_BM:THC8	0.62	11.92	0.53	3.88	[51]
P3HT:PC_61_BM:SiPc	0.58	11.1	0.65	4.13	[52]
P3HT:PC_61_BM:TIPS-pentacene	0.61	10.86	0.62	4.13	[53]
P3HT:PC_61_BM:PCDPP4T	0.53	11.1	0.59	3.5	[54]
P3HT:PC_61_BM:ZnPc	0.62	12.6	0.68	5.3	[55]
P3HT:PCBTDPP:PC_61_BM	0.64	16.15	0.50	5.28	This work
